# A Pecan-Rich Diet Improves Cardiometabolic Risk Factors in Overweight and Obese Adults: A Randomized Controlled Trial

**DOI:** 10.3390/nu10030339

**Published:** 2018-03-11

**Authors:** Diane L. McKay, Misha Eliasziw, C. Y. Oliver Chen, Jeffrey B. Blumberg

**Affiliations:** 1 Antioxidants Research Laboratory, Jean Mayer USDA Human Nutrition Research Center on Aging at Tufts University, Boston, MA 02111, USA; oliver.chen@tufts.edu (C.Y.O.C.); jeffrey.blumberg@tufts.edu (J.B.B.); 2 Department of Public Health and Community Medicine, Tufts University School of Medicine, Boston, MA 02111, USA; misha.eliasziw@tufts.edu

**Keywords:** pecan, tree nut, diabetes, heart disease, obesity, metabolic syndrome, insulin, HOMA-IR, glucoregulation, polyphenol

## Abstract

Evidence from observational and intervention studies has shown a high intake of tree nuts is associated with a reduced risk of cardiovascular disease (CVD), mortality from type 2 diabetes (T2DM), and all-cause mortality. However, there is limited data regarding their effects on indicators of cardiometabolic risk other than hypercholesterolemia, and little is known about the demonstrable health benefits of pecans (*Carya illinoensis* (Wangenh.) K.Koch). We conducted a randomized, controlled feeding trial to compare the effects of a pecan-rich diet with an isocaloric control diet similar in total fat and fiber content, but absent nuts, on biomarkers related to CVD and T2DM risk in healthy middle-aged and older adults who are overweight or obese with central adiposity. After 4 weeks on a pecan-rich diet, changes in serum insulin, insulin resistance (HOMA-IR) and beta cell function (HOMA-β) were significantly greater than after the control diet (*p* < 0.05). Pecan consumption also lowered the risk of cardiometabolic disease as indicated by a composite score reflecting changes in clinically relevant markers. Thus, compared to the control diet, the pecan intervention had a concurrent and clinically significant effect on several relevant markers of cardiometabolic risk.

## 1. Introduction

Cardiovascular diseases (CVD) are a leading cause of death worldwide, and a major concern for global health [1]. Within the past decade, there has also been a dramatic increase in diet-related chronic diseases related to CVD risk, i.e., diabetes, obesity, and hypertension, in both industrialized and developing nations, thus adding to this global burden [2]. Many factors contribute to an increased risk of developing CVD, particularly the presence of obesity, as the active metabolism of adipose tissue induces metabolic changes, e.g., increased production of reactive oxygen species, oxidative stress, and inflammation, leading to type 2 diabetes mellitus (T2DM), arterial hypertension, and dyslipidemia. Thus, metabolic biomarkers, such as blood glucose, insulin resistance, and lipid profile, as well as indicators of inflammation and oxidative stress, are important indicators of risk. Risk markers are important indicators of subclinical disease and a valuable tool for CVD monitoring and prevention [3].

A growing body of evidence from observational and intervention studies has shown that a high intake of nuts is associated with a reduced risk of CVD development, all-cause mortality, and mortality from diabetes [4,5]. Indeed, a nut-containing diet low in saturated fat and cholesterol, while high in poly- and monounsaturated fatty acids, has a beneficial effect on plasma lipids and lipoproteins when compared with either a low fat or average American diet. However, the low density lipoprotein (LDL) cholesterol-lowering response shown in these studies is greater than expected based on equations derived from dietary fatty acid profiles, and may not be solely due to the fatty acid composition of nuts [6]. Other bioactive compounds present in nuts, including micronutrients, fiber, and phytochemicals, may also contribute to their cardio-protective effect by reducing inflammation, improving vascular reactivity as well as fasting glucose and insulin sensitivity, and by lowering oxidative stress status [7,8,9,10,11,12,13,14]. Therefore, well-controlled studies focusing on the additive and/or synergistic effects of the nut constituents present in the whole food matrix, and the degree to which they confer a health benefit and prevent chronic disease, are warranted.

While most randomized clinical trials (RCT) to date have focused on the beneficial effect of nuts on plasma lipids in subjects with hypercholesterolemia, and some on glycemic control in T2DM patients [15], limited data are available regarding their effects on other indicators of CVD risk, i.e., metabolic syndrome (MetS), elevated blood pressure, endothelial function, and in other at-risk populations. Furthermore, in most RCT of nuts, subjects were counseled on how to incorporate the prescribed quantity of nuts into their usual diet. This approach cannot control for inter-individual variability in the background diet, or fully account for the intake of other components in foods and beverages that can also influence CVD and T2DM risk, e.g., antioxidants, fatty acids, and fiber.

Despite their use for centuries by Native American tribes in the U.S. and Mexico, very little is known about the putative health benefits of pecans (*Carya illinoensis* (Wangenh.) K.Koch). To date only a small number of studies have examined the effects of this tree nut on human health [16,17,18,19]. Morgan & Clayshulte (2000) and Rajaram et al. (2001) focused primarily on lipid profile changes in normal to mildly overweight young and middle-aged adults with low to moderately elevated serum cholesterol levels, while Haddad et al. (2006) and Hudthagosol et al. (2011) focused on markers of antioxidant capacity and oxidative stress. No studies have assessed the effects of pecan consumption on other markers of cardiometabolic risk, i.e., fasting blood glucose or insulin, blood pressure, inflammation, and endothelial function, or in subjects who might be more predisposed to this condition. Exploring the potential health benefits of pecans is worthwhile as they are a good source of mono-(oleic acid) and polyunsaturated (linoleic acid) fats and fiber, but also due to their profile of phytochemicals (total phenols, proanthocyanidins, hydrolysable tannins, flavonoids, phenolic acids), fat-soluble bioactive components (phytosterols, sphingolipids, tocols), and essential minerals (magnesium, manganese, zinc) [20,21].

In order to address this gap, we conducted a randomized, controlled feeding study to compare the effects of a pecan-rich diet with an isocaloric control diet similar in total fat and fiber content, but absent nuts, on biomarkers related to CVD and T2DM risk, including indices of insulin sensitivity, oxidative stress, antioxidant activity, inflammation, and endothelial function, in healthy middle-aged and older adults who are overweight or obese with central adiposity. Obesity, particularly intra-abdominal obesity, especially in older adults, predisposes people to several modifiable risk factors of CVD and T2DM, i.e., cardiometabolic risk. We hypothesize the daily consumption of a diet including whole pecans, in an amount compatible with the established U.S. Food and Drug Administration (FDA) qualified health claim for tree nuts [22] will attenuate changes in biomarkers associated with cardiometabolic disease risk in this population.

Conducting a controlled feeding trial of pecans in overweight or obese subjects with central adiposity will not only allow us to confirm their lipid-lowering properties in this at-risk population, it will expand our knowledge of their effects on other biomarkers of CVD and T2DM risk and further ascertain the degree to which pecans may help maintain health and reduce the risk for chronic disease. Given the high prevalence of overweight and obese adults in the U.S. [23] and worldwide [24], the results of this study may be relevant to a broad segment of the adult population.

## 2. Materials and Methods

### 2.1. Subject Eligibility

**Figure 1:**
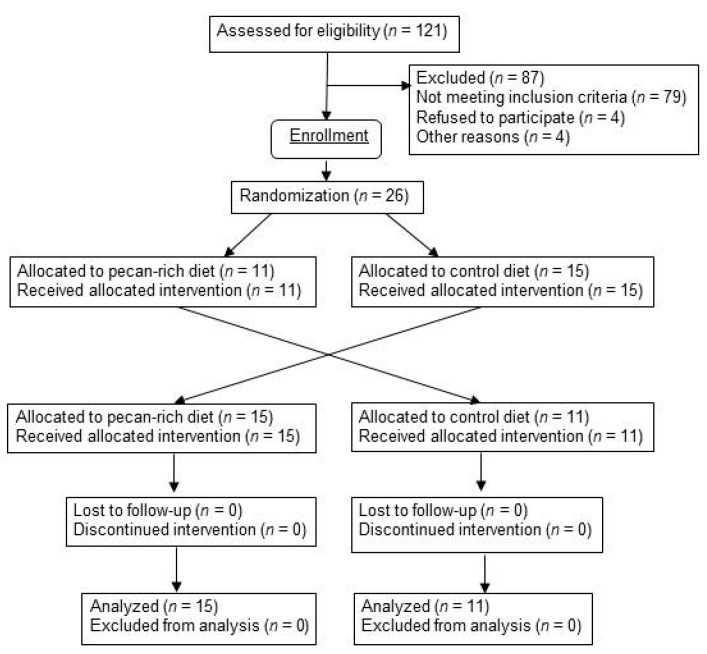
Study recruitment flow diagram.

Twenty six (26) metabolically at-risk, nonsmoking men and postmenopausal women, age ≥ 45 year, who are overweight or obese (body mass index (BMI) 25–35 kg/m^2^) with central adiposity (waist:hip ratio >0.80 for women, >0.90 for men, and waist:height ratio >0.5) were recruited from the Boston area. Postmenopausal status in women was defined as the absence of menses for ≥1 year. The exclusion criteria used to screen for eligibility included: (a) presence of CVD; (b) use of estrogen, with or without progesterone; (c) use of medications known to affect lipid metabolism; (d) use of medications known or suspected to influence blood pressure (BP); (e) gastrointestinal diseases and conditions or medications influencing gastrointestinal absorption; (f) chronic kidney disease; (g) endocrine disorders including diabetes and untreated thyroid disease; (h) rheumatologic disorders; (i) active treatment for cancer of any type (except basal cell carcinoma) ≤1 year; (j) regular use of oral steroids; (k) regular use of anti-inflammatory agents (prescribed or over-the-counter (OTC)); (l) inability to discontinue or refrain from aspirin, non-steroidal anti-inflammatory drugs (NSAID), or acetaminophen use for 72 h prior to and during testing visits; (m) systolic blood pressure (SBP) >150 mmHg and/or diastolic blood pressure (DBP) >95 mmHg; (n) regular use of any dietary supplements within ≤30 days; (o) usual daily ethanol intake of ≥2 drinks; (p) cigarette smoking and/or nicotine replacement use; (q) allergy to nuts of any kind; (r) frequent nut consumption (>142.8 g/week) or inability to refrain from consuming all nuts and nut products within ≤30 day; (s) unwillingness or inability to consume animal-based foods; and (t) laboratory blood or urine biochemistries outside of normal ranges. Thirty subjects were recruited and followed from March 2014 to May 2016. Four subjects withdrew from the study prior to starting the intervention because they were either no longer interested in participating or unwilling to adhere to the required protocol (Figure 1). The study design was approved by the Institutional Review Board of Tufts University Health Sciences Campus and Tufts Medical Center. All participants signed a written informed consent agreement before participating. This study was registered with the public registry ClinicalTrials.gov (ID #NCT 01950806).

### 2.2. Study Design and Intervention

A randomized, blinded, placebo-controlled, crossover trial was conducted to determine whether the daily consumption of a pecan-rich test diet (15% of total calories) for 4 weeks improves selected biomarkers related to CVD and T2DM risk, i.e., glucoregulation and insulin resistance (fasting insulin, fasting glucose, calculated Homeostasis Model of Assessment (HOMA) for insulin resistance (HOMA-IR) and beta-cell function (HOMA-B)), oxidative stress (susceptibility of LDL to Cu^+2^-induced oxidation (LDLox)), antioxidant markers (total thiols, total phenolics, α- and γ-tocopherol, inflammation (high-sensitivity C-reactive protein (hsCRP)), and endothelial function (E-selectin, endothelin-1), when compared with a control diet absent nuts and matched for total fat and fiber content. Secondary outcomes included plasma lipids (total cholesterol, triglycerides (TG), very low density lipoprotein (VLDL), low density lipoprotein (LDL), high density lipoprotein (HDL)), red blood cell (RBC) fatty acids, and blood pressure (SBP, DBP).

This 12 week trial included a two-week run-in period, two four-week feeding periods (Period I and Period II) during which all meals were provided to the subjects, and a two-week washout period between Periods I and II. During the run-in and washout periods, eligible subjects were counseled to consume a control diet resembling the average American diet, i.e., low in fruit, vegetables, fiber, and *n*-3 fatty acids, and high in nutrient-poor, calorie-dense foods. During Period I, subjects were randomized to receive either the control diet alone or the control diet with whole pecans (test diet) incorporated as a major source of energy, and asked to consume only the meals/snacks provided to them. In Period II subjects received the opposite diet they were assigned during Period I. Fasting blood samples, 24 h urine samples, and clinical measures were collected at the baseline and end of Periods I and II.

Randomization was stratified by gender according to a computer-generated list. Study personnel were blinded to the treatment assignment for the duration of the intervention and sample analysis. The only exception was the study dietitian who was responsible for distributing the dietary instructions, and meals to eligible subjects at randomization, and assessing compliance. During each visit, subjects reported to the Metabolic Research Unit (MRU) at the Jean Mayer USDA Human Nutrition Research Center on Aging (HNRCA) at Tufts University after fasting for 12 h. At each visit, subjects were queried regarding interval changes in health, as well as use of prescription medications, alcohol, tobacco, and dietary supplements. During Periods I and II, subjects were asked to return twice every week to pick up their supply of meals and drop off their empty containers and unused food. All meals were prepared in the MRU kitchen under the direction of the study dietitian.

### 2.3. Study Diets

The control diet resembled a typical American diet, i.e., low in fruit, vegetables, fiber, and *n*-3 fatty acids, and high in nutrient-poor, calorie-dense foods. The pecan-rich intervention diet included pecans isocalorically substituted for 15% of the total energy (~42.5 g/2000 kcal). The calorie content of each diet was adjusted to meet the energy needs of each subject while maintaining their initial body weight. Energy needs were calculated using the Harris-Benedict equation. The macronutrient composition of the control diet (Table 1) reflected the mean nutrient intake of U.S. adults ≥50 year according to the last What We Eat in America, National Health and Nutrition Examination Survey (NHANES) 2005–2006 survey data [25]. The total number of fruit and vegetable servings reflected the geometric mean frequency of daily consumption according to the U.S. Behavioral Risk Factor Surveillance System [26]. Caffeinated coffee and tea were limited to ≤2 cups/day as higher levels of consumption (≥4 cups/day) have been associated with increases in plasma adiponectin, an adipokine [27]. Subjects were also be instructed to limit their intake of non-caffeinated coffee and tea, as well as other plant-based beverages (e.g., herbal tea), and eliminate their intake of alcohol for the duration of the study.

### 2.4. Sample Collection and Preparation

Blood samples for the analysis of ferric reducing ability of plasma (FRAP), LDLox, lipids, fatty acids, phenols, and tocopherols were collected in ethylenediaminetetraacetic acid (EDTA)-containing evacuated tubes and centrifuged within 15 min of drawing (1000× *g*, 15 min, 4 °C) with a SUR-Sep cap (Organon Teknika, Durham, NC, USA). Blood samples for the remaining analytes were collected in serum separator tubes and processed similarly. Plasma samples for the analysis of LDLox were prepared by adding 111 µL of 6% sucrose solution to 1 mL plasma, and stored at −80 °C for ≤8 weeks before analysis. All samples were stored at −80 °C until analysis. For the fatty acid analyses, packed red blood cells (RBC) were washed with saline and butylated hydroxytoluene added at 0.4 mg/mL to prevent oxidation. Sample aliquots were stored in 2 mL Nunc tubes (Vanguard Cryotubes, Neptune, NJ, USA) at −80 °C. All samples for each participant were analyzed within the same run for every assay performed.

### 2.5. Biochemical Analyses

#### 2.5.1. Glucoregulation and Insulin Sensitivity

Serum glucose was measured by an enzymatic couple method using an AU400 clinical chemistry analyzer (Beckman Coulter, Inc., Brea, CA, USA) according to the manufacturer’s instructions, with intra- and inter-assay coefficient of variation (CV) of 2.0% and 3.2%, respectively. Serum insulin was measured using a solid phase sandwich enzyme linked-immuno-sorbent assay kit procedure, (Invitrogen Human Insulin ELISA kit, Camarillo, CA, USA) according to the manufacturer’s instructions, with intra- and inter-assay CV of 5.4% and 8.5%, respectively. Insulin sensitivity was assessed using the HOMA-IR based on the formula: (insulin × glucose)/22.5 with insulin expressed as U/mL and glucose as mmol/L [28]. Beta cell function was assessed using the HOMA-B based on the formula: (20× insulin)/glucose −3.5) [29].

#### 2.5.2. Oxidative Stress

The ex vivo resistance of LDL to Cu^2+^-induced oxidation was determined by monitoring the formation of conjugated dienes at 37 °C over 3 h with a Shimadzu UV1601 spectrophotometer (Shimadzu Scientific Instruments, Inc., Columbia, MD, USA) at an absorbance of 234 nm according to Chen et al. (2007) [11]. The results are expressed as lag time (min).

#### 2.5.3. Antioxidant Markers:

Total thiols (-SH moieties) in plasma were determined according to the spectrometric method of Hu [21]. The total phenolic content of plasma was determined by the Folin-Ciocalteu reaction according to Singleton et al. (1999) [30] with the data expressed as µmol gallic acid equivalents (GAE)/L. Plasma α- and γ-tocopherols were quantified using an ultra-high pressure liquid chromatography (UHPLC)-fluorescence detection method according to Liu et al. (2011) [31].

#### 2.5.4. Inflammation and Endothelial Function

High sensitivity CRP (hsCRP) in serum was measured by a solid-phase, two-site chemiluminescent immunometric assay using the IMMULITE 2000 (Siemens Healthcare Diagnostics, Los Angeles, CA, USA). The intra- and inter-assay CVs were 3.0% and 5.0% respectively. E-selectin and endothelin in serum were measured separately using quantitative solid phase sandwich enzyme-linked immunosorbent assay kit procedures (Quantikine^®^ Human sE-Selectin/CD62E Immunoassay and Quantikine^®^ Endothelin-1 Immunoassay) from R&D Systems, Inc, (Minneapolis, MN, USA) on the BioTek Instrument ELx 808 Microplate Reader. The intra- and inter-assay CVs were 5.8% and 7.9% for E-selectin, and 3.0% and 6.3% for endothelin.

#### 2.5.5. Lipids and Fatty Acids

Plasma concentrations of total cholesterol, LDL, HDL, and triglycerides were determined with a clinical chemistry analyzer (Olympus AU400, Center Valley, PA, USA) according to the manufacturers’ instructions. RBC fatty acid profiles were quantified using a gas chromatography method as previously described [32,33]. Peaks of interest were identified by comparison with authentic fatty acid standards (Nu-Check-Prep, Elysian, MN, USA) and the data expressed as molar percentage (mol%) proportions of fatty acids relative to the internal standard.

### 2.6. Clinical Assessments

#### 2.6.1. Anthropometric Measurements

During the study screening visit, all anthropometric measurements were taken while subjects wore a hospital gown and undergarments only. The same apparatus and equipment were used to assess weight, height, waist, and hip circumference measurements in all subjects. Waist and hip circumference was assessed according to the standardized method used in NHANES III [34]. If duplicate measures of both waist and hip circumference were within 0.5 cm, the values were averaged. If the variation was >0.5 cm, a third measurement was taken.

Although there is currently a lack of consensus on the most appropriate cut-off values for assessing abdominal obesity [35], as well as a great disparity in the optimal cut-off points both within and between ethnic groups [36], the cut-off values for inclusion in our study are justified. There is agreement that both waist:height and waist:hip ratios are better predictors of abdominal fatness than BMI alone [36,37]. Ashwell and Hseih (2005) [38] argue that a cut-off value of waist/height ratio ≥0.50 indicates increased risk for men and women among several different ethnic groups.

#### 2.6.2. Blood Pressure Measurements

SBP and DBP were measured at the brachial artery by use of an automated BP device (Dinamap ProCare 220, GE HealthCare, Milwaukee, WI, USA) to minimize investigator variability and terminal digit bias. Recommended procedures for BP measurement, with all possible efforts to minimize common pitfalls, were observed [39]. A standardized protocol was followed for each BP measurement: The appropriate cuff size for each participant was determined, and the same arm and cuff were used for all measurements. Each participant reported to the HNRCA at the same time of day for each measurement, and sat in a quiet environment in a comfortable chair, with feet on the floor, for 15 min after which BP was measured with the arm at heart level. The BP measurement was then repeated every 5 min for the next 15 min. Values for these 3 determinations of SBP, DBP, and mean arterial pressure (MAP) were averaged.

### 2.7. Statistical Analyses

Statistical analyses were performed using SAS version 9.4 (SAS Institute Inc., Cary, NC, USA). For each outcome variable, the mean change was compared between the intervention and control arms using a standard approach for analyzing a crossover design; a linear mixed model that included factors to assess period and carryover effects, as well as adjusting for the baseline value of the variable of interest. Unless otherwise noted, results are presented as mean ± standard error (SE) with respective *p* values for within and between group differences. *p*-values < 0.05 were considered statistically significant.

Subgroup analyses were performed by including a cross-product term in the linear mixed model to assess treatment effect modification by the following variables: age (<62 vs. ≥62 year), sex (males vs. females), BMI (<30 vs. ≥30 kg/m^2^), waist:hip (<1 vs. ≥1), glucose (≤100 vs. >100 mg/dL), total cholesterol (<200 vs. ≥200 mg/dL), LDL cholesterol (<130 vs. ≥130 mg/dL), and SBP (<120 vs. ≥120 mmHg).

An additional analysis was also performed to assess the effect of the intervention on the simultaneous change of five selected cardiometabolic risk factors (insulin, glucose, total cholesterol, TG, and LDL) in the following manner:

First, each of the five cardiometabolic risk factors were normalized to yield *z*-scores using means ($\bar{x}$) and standard deviations (𝒔) derived from their reference ranges:

$$\mathrm{insulin},\;\bar{x}=7.7\;\mathrm{uIU}/\mathrm{mL},\;𝒔=3.2\;\mathrm{uIU}/\mathrm{mL};\;\mathrm{glucose},\;\bar{x}=87.5\;\mathrm{mg}/\mathrm{dL},\;𝒔=8.9\;\mathrm{mg}/\mathrm{dL};\;\mathrm{total}\;\mathrm{cholesterol},\;\bar{x}=180.0\;\mathrm{mg}/\mathrm{dL},\;𝒔=25.5\;\mathrm{mg}/\mathrm{dL};\;\mathrm{TG},\;\bar{x}=126.5\;\mathrm{mg}/\mathrm{dL},\;𝒔=41.6\;\mathrm{mg}/\mathrm{dL};\;\mathrm{LDL},\;\bar{x}=105.0\;\mathrm{mg}/\mathrm{dL},\;𝒔=10.2\;\mathrm{mg}/\mathrm{dL}.$$

Second, change in each cardiometabolic risk factor level was calculated by subtracting their corresponding baseline *z*-score from their follow-up *z*-score. Finally, a multivariate multilevel regression model (i.e., linear mixed model) was used to simultaneously assess change in all five *z*-scores between the intervention and control groups, and included factors to assess period and carryover effects, as well as adjusting for the baseline value of the *z*-scores. In the regression model, the outcome variable consisted of all five cardiometabolic risk factor *z*-score changes for each participant at each period, yielding a total of 260 records of data. A compound symmetry covariance structure was used in the regression model to account for the interdependence among the cardiometabolic risk factors arising from the same participant. The resulting regression model yielded a single estimate of the mean difference in z-score change between diets.

## 3. Results

### 3.1. Subject Characteristics

Table 2 presents the baseline characteristics of the 26 subjects who completed the trial. Of these subjects, 19 (73%) had elevated LDL (≥100 mg/dL, 30% were ≥130 mg/dL), 17 (65%) had low HDL (<60 mg/dL, 50% were <50 mg/dL), 15 (58%) had elevated SBP (≥120 mmHg), 13 (50%) had elevated fasting blood glucose levels (≥100 ng/dL), 12 (46%) had elevated DBP (≥80 mmHg), and 10 (38%) had elevated total cholesterol (≥200 mg/dL) and/or were obese (BMI ≥ 30 kg/m^2^). No untoward effects related to the intervention were reported.

### 3.2. Main Outcomes

The study outcomes are presented in Table 3. After four weeks on a pecan-rich diet, changes in subjects’ serum insulin, insulin resistance (HOMA-IR) and beta cell function (HOMA-β) were significantly greater than on the control diet (*p* = 0.024, *p* = 0.037, *p* = 0.021, respectively). Although both total and LDL cholesterol were lower following the pecan diet, their magnitude of change compared with the control diet was of borderline significance (*p* = 0.056, *p* = 0.067, respectively). Other markers of cardiometabolic risk, including serum glucose, SBP and DBP, were also observed to be lower with the pecan diet; however, neither the within- nor between-group differences reached statistical significance. No significant changes in body weight were observed over the study period (*p* = 1.00).

### 3.3. Composite Index

The analysis of the aggregate change of five selected cardiometabolic risk factors yielded a mean decrease of 0.14 standard deviations (SD) with the pecan-rich diet and an increase of 0.25 SD with the control diet (mean difference of 0.39 SD between groups, *p* = 0.009) (Table 4). Thus, when compared with the control diet, the pecan intervention had a concurrent and statistically significant effect on several clinically relevant markers of cardiometabolic risk.

### 3.4. Antioxidants, Oxidative Stress, Inflammation, and Endothelial Function

Table 5 presents the observed effects of the intervention diets on selected biomarkers associated with risk of T2DM and CVD. No statistically significant differences between groups were observed in the level of tocopherols, total phenolics, biomarkers of antioxidant capacity, oxidative stress, inflammation, or endothelial function. Pecans are high in γ-tocopherol and, as expected, levels of this form of vitamin E increased with pecan consumption, while levels of α-tocopherol decreased. When adjusted for total cholesterol, these changes in tocopherol levels, i.e., the tocopherol:cholesterol ratios, also did not differ between groups. Total phenolics increased similarly in both groups, perhaps due to the addition of wheat bran fiber to the control diet in order to adjust fiber levels accordingly. A slightly longer lag time for oxidation of LDL was observed following the pecan diet when compared with the control, indicating a protective effect, although this change did not reach statistical significance.

### 3.5. RBC Fatty Acids

Table 6 presents the changes observed in the most predominant fatty acids present in subjects’ red blood cells. Compared with the control diet, levels of total monounsaturated fatty acids (MUFA) and oleic acid, the predominant fatty acid present in pecan oil, were significantly higher (*p* = 0.0099, *p* = 0.0003), and palmitic acid, a common saturated fat, were lower (*p* = 0.0489) with the pecan diet. These changes indicate a high degree of compliance with the study protocol. Although the pecan diet raised the level of *n*-6 polyunsaturated fatty acids (PUFA) above baseline (*p* = 0.01), and reduced *n*-3 PUFAs with both diets (*p* = 0.02 for pecan, *p* = 0.03 for control), their between-group differences were not significant.

### 3.6. Subgroup Analyses

Several factors modified the effectiveness of the pecan vs. control diet, and they are summarized in Table 7. These data indicate that the pecan diet may be more effective in males than in females with insulin problems, which is consistent with the evidence showing men are at higher risk for CVD. A stronger association between nut intake and CVD risk in men has also been recently noted [4]. The reduction in LDL cholesterol with the pecan diet was more pronounced in subjects with higher fasting glucose levels, while the reduction in total cholesterol was greater in those with lower baseline LDL levels. Interestingly, the effectiveness of pecans on insulin secretion was greater among subjects with higher baseline SBP. Although the number of subjects in each comparison subgroup was relatively small, the study was well-controlled and, thus, the results of this analysis may prove useful in directing future research.

## 4. Discussion

This is the first reported placebo-controlled feeding trial to examine the effects of pecans on cardiometabolic risk factors other than blood lipid levels, and in a population of middle-aged and older adults who are predisposed to developing CVD and/or T2DM. We determined the daily consumption of ~1.5 oz (42.5 g) of pecans, when incorporated into a typical American diet, effectively improved insulin resistance, fasting insulin, and beta cell function in overweight and mildly obese adults with central adiposity. Obesity, coupled with insulin resistance increases insulin demand and hyperfunction of pancreatic beta cells resulting in their eventual dysfunction [40]. Our results suggest pecans may help curtail this process in overweight and obese adults by improving insulin sensitivity, thus reducing demand and suppressing overproduction.

Pecan consumption lowered the risk of cardiometabolic disease as indicated by a composite score reflecting a significant change in clinically relevant markers, i.e., blood lipids and glucoregulation. Using a composite score to concurrently analyze changes in several markers is not unique. However, our method, which used reference ranges to normalize the markers, demonstrates how appropriately aggregating similar markers increases statistical power to detect a difference between groups.

Several trials have reported the effects of tree nuts other than pecans on measures of blood glucose control in diabetic patients or in those with MetS. Casa-Agustench et al. (2011) [41] reported similar reductions in fasting insulin (−2.60 µU/mL (95% confidence interval (CI): −4.62, −0.59)) and HOMA-IR (−0.72 (95% CI: −1.28, −0.16)) (*p* < 0.05 for both) in a parallel, controlled trial of 50 MetS patients supplemented with 30 g/day mixed nuts (15 g walnuts, 7.5 g each of almonds and hazelnuts) for 12 weeks. Hernandez-Alonso et al. (2014) [42] observed greater reductions in fasting glucose, insulin, and HOMA-IR in a crossover study of 54 prediabetic subjects who consumed 57 g/day pistachios for 4 months vs. an isocaloric diet matched for protein, fiber and saturated fat. However, in a pooled analysis of 450 patients with diabetes, Viguiliouk et al. (2014) [43] reported lower fasting glucose (mean difference (MD) = −0.15 mmol/L (95% CI: −0.27, −0.02); *p* = 0.03), but no effect on fasting insulin or HOMA-IR with a median dose of 56 g/day for 8 weeks. In a separate pooled analysis of 2226 subjects who were either healthy, or had dyslipidemia, MetS or T2DM, Blanco Mejia et al. (2014) [44] reported tree nut interventions of similar median duration and dose (49.3 g/day) were also effective in lowering fasting blood glucose (MD = −0.08 mmol/L (95% CI: −0.16, −0.01); *p* < 0.05) by an amount similar to that observed in our study (−0.08 mmol/L change in glucose), when compared with a control diet; however, insulin was not included in their analysis. The findings from our study add to this literature by confirming that, similar to other tree nuts, regular consumption of pecans, in an amount that is readily incorporated into the diet, may help control blood glucose in at-risk individuals by affecting the release and action of insulin.

Potential mechanisms for the observed effects of nuts on blood glucose control include increased cell membrane permeability due to their high concentration of unsaturated fatty acids; inhibition of enzymes involved in carbohydrate digestion and absorption; reduced levels of aspartate aminotransferase, an enzyme associated with insulin resistance in the obese; and delayed digestion and postprandial absorption of glucose due to their high fat, fiber, and phenolic content [3]. Indeed, when eaten alone nuts have been shown to have a minimal effect on raising postprandial glucose in acute feeding studies, and may blunt the glycemic response to a high carbohydrate meal [15]. However, since the total calories from carbohydrate, protein, and fat were identical in our pecan and control diets, the displacement of carbohydrates is unlikely to be the primary mechanism of action. Given the increase in RBC MUFA and oleic acid concentrations following pecan consumption, a more plausible mechanism involves improved cell membrane fluidity and increased permeability to insulin [45].

Other potential mechanisms may involve the anti-inflammatory and antioxidant properties of pecan polyphenols as evidence suggests polyphenol-rich diets reduce T2DM risk perhaps by inhibiting intestinal glucose absorption, protecting pancreatic beta cells against glucose toxicity, and/or suppressing the release of glucose from liver storage and peripheral tissues [46,47,48]. The hydrolysable tannins present in pecans, and their derivatives ellagic acid and the urolithins in particular, may ameliorate the effects of chronic metabolic disease by decreasing the expression of genes related to chronic inflammation, but a definitive mechanism of action has not yet been established [49].

The borderline reductions in total cholesterol and LDL cholesterol observed in our study may be attributable to the lower dose compared with other tree nut trials, and/or to the higher prevalence of obesity among our subjects. In their meta-analysis of 61 RCT, Del Gobbo et al. (2015) [50] determined doses ≥60 g/day tree nuts are needed to see a reduction in LDL. Only two other pecan RCT have examined the effects of this nut on lipid profiles. Both studies used a dose above this level, and included non-obese, generally younger adults [16,17]. In a parallel design study, after 8 weeks Morgan and Clayshulte (2000) [16] reported 3 and 6% reductions in total cholesterol and LDL, respectively, in 19 healthy, normal weight (mean BMI 24 kg/m^2^) subjects, age < 50 year, who were on self-selected diets including 68 g/day pecans, while in control subjects total cholesterol and LDL increased 7 and 11%, respectively (*p* < 0.05 between groups). Using a crossover design study, Rajaram et al. (2001) [17] reported total cholesterol and LDL reductions of 11 and 17%, respectively, in 23 healthy subjects, age 25–55, with BMI < 30 kg/m^2^, who were given 72 g/day pecans for 4 weeks, compared with reductions of 5 and 7%, respectively, following a low-fat Step 1 diet (*p* < 0.05 between groups). In our study, total cholesterol and LDL reductions were 0.5 and 1%, respectively, with 45 g/day pecans, and without pecans these respective levels increased by 4 and 5%. Although our dose was ~30% less than these earlier pecan studies, most of our subjects were >60 year and ~40% were obese. In a pooled analysis of RCT that examined the effects of nuts on lipid lowering, Sabate et al. (2010) [51] observed a blunted response to nut consumption in obese subjects, which is consistent with our findings. Furthermore, most studies conducted in individuals with MetS report nut intake has little to no effect on LDL [52]. Among obese subjects dietary interventions were reported to be less effective in lowering cholesterol [53], perhaps due to reduced intestinal cholesterol absorption seen in obese and insulin-resistant subjects [54]. Although not measured in our study, apolipoproteins may be more indicative of atherogenic activity than LDL alone [55], and nuts may be particularly effective in lowering levels among diabetics [50]. Indeed, pecans were shown to upregulate the hepatic expression of apolipoprotein B and LDL receptor mRNAs in rats fed a high fat diet supplemented with whole pecans [56]. However, the effects of pecan consumption on apolipoprotein levels in humans have not been reported.

The lack of a significant effect on measures of BP and inflammation might also be related to dose and subject characteristics, as well as specific nut components. In their meta-analysis, Del Gobbo et al. (2015) similarly reported no significant effects of tree nuts on BP or inflammation, even with doses ≥60 g/day, while a meta-analysis by Mazidi et al. (2016) [57] found no significant effect of nuts on CRP or other markers of inflammation with doses up to 128 g/day. In an analysis of 1652 adults from 21 RCT, Mohammadifard et al. (2015) [58] also found overall nut consumption had no effect on either SBP or DBP, except for subjects in whom T2DM was absent. Among all the tree nuts considered in their analysis, pistachios appeared to have the strongest BP-lowering effect. Similar to pecans, pistachios are high in MUFAs, as well as total phenols, proanthocyanidins, and flavonoids (including catechins), but unlike pecans and other nuts, pistachios are high in carotenoids (lutein), stilbenes, chlorophyll, and isoflavones [20,59]. Whether differences in these, or other nut components, are responsible for their reported effect on BP has yet to be determined. Furthermore, no RCT to date have examined the effects of any type of tree nut on BP in pre- or hypertensive subjects.

Changes in measures of antioxidant capacity and oxidative stress, factors related to cardiometabolic risk, were also favorable but not significant following pecan consumption. Similar to the study conducted by Haddad et al. (2006) in which subjects were given 72 g/day pecans for 4 weeks, we observed comparably proportional changes in plasma γ- and α-tocopherol levels, albeit to a lesser degree. They also noted no statistically significant changes in antioxidant status, measured as FRAP, but did note a reduction in lipid peroxidation measured as plasma thiobarbituric acid reactive substances. Similarly, we noted a reduction in the susceptibility of LDL to lipid peroxidation as did Hudthagosol et al. (2011). Following an acute dose of 90 g pecans Hudthagosol et al. (2011) also noted significant increases in plasma γ-tocopherol and antioxidant activity, measured as hydrophilic- and lipophilic-Oxygen Radical Absorbance Capacity, in healthy, young non-overweight adults. One reason why the changes observed in our study were not significant may be that the effects of nuts on plasma measures of oxidative stress are less pronounced in subjects with MetS. Lopez-Uriarte et al. (2010) [60] reported no changes in several plasma markers in a RCT of 50 MetS patients given 30 g/day nuts for 12 weeks, although oxidative DNA damage was significantly reduced. In a systematic review of studies that examined the effects of dietary polyphenols in MetS subjects, Amiot et al. (2016) [47] also noted that not all RCT reported reductions in oxidative stress in this population.

While the antioxidant capacity of most polyphenol-rich foods, including pecans, is typically high when measured prior to consumption [61], these compounds are poorly absorbed and extensively metabolized via Phase I and II biotransformations during digestion and prior to tissue distribution. The resulting metabolites may act via several different mechanisms at the cellular and subcellular level, many of which are not necessarily reflected by standard measures of plasma antioxidant capacity. In fact, the physiological effects of polyphenols appear mediated largely by their metabolites acting to modulate cell cycling, cell proliferation, detoxification, and inflammation mediated via cell signal transduction pathways [62]. Few studies have examined the effects of pecans in this capacity. Thus, more research is required to ascertain the specific effects of pecan polyphenols and their potential mechanisms of action.

The strengths of our study include a controlled crossover feeding trial design to account for variations in background diet and inter-individual variation; similar total fat and fiber contents between the study diets to determine the effects attributable to the specific types of fat and polyphenols present in pecans; and high compliance with the study protocol as indicated by increased levels of RBC oleic and linoleic acids, the predominant MUFA and PUFA present in pecans, following the respective feeding period. Pecans were also incorporated in a reasonable quantity that can be readily achieved in the usual diet. Furthermore, using a background diet that closely represented a typical eating pattern for U.S. adults, allowed us to determine the benefits of a simple substitution towards the goal of a more healthful overall diet. Limitations to our study include the small sample size and relatively short duration, both of which reduced our ability to detect as statistically significant the smaller, more subtle changes that may occur with longer term, regular consumption in a larger population. Our cohort was also relatively well nourished as indicated by their baseline nutrient levels and markers of antioxidant status. If the background test diet provided fewer plant-based foods than their usual diet, this may have affected our ability to detect larger changes in markers of antioxidant status, inflammation and endothelial function.

## 5. Conclusions

Elevated blood glucose levels and insulin resistance are major risk factors for cardiometabolic disease and lifestyle modification, including dietary change, is the recommended first-line approach to disease prevention and management. The dietary change assessed in this study, displacing a portion of the saturated fat in a typical American diet and enhancing phytochemical intake with a handful of whole pecans daily, can protect adults at risk for developing CVD and T2DM due to their age, overweight status, and body fat distribution. Further investigation of nut consumption on the prevention of cardiometabolic risk in at-risk populations is warranted, as are more studies examining the potential benefits and associated mechanisms of action related to regular pecan consumption.

## Figures and Tables

**Table 1:** Estimated nutrient composition of the study diets ^1^.

Component	Control Diet	Pecan Diet
Energy (kcal)	2024	2027
Carbohydrate (%kcal)	48.3	48.2
Protein (%kcal)	15.6	16.4
Total fat (%kcal)	36.0	35.3
Saturated fat (%kcal)	13.6	9.1
MUFA (%kcal)	10.8	14.8
PUFA (%kcal)	8.4	8.6
PUFA *n*-6 (g)	17.1	18.7
PUFA *n*-3 (g)	1.7	1.3
Cholesterol (mg)	256	249
Fiber (g)	18.2	18.0
Fruit (# servings)	1.1	1.1
Vegetables (#servings)	2.3	2.3

^1^ Average of 3-day meal cycle; values obtained from the USDA National Nutrient Database for Standard Reference (USDA 2016) [21]. Abbreviations: MUFA, monounsaturated fatty acids; PUFA polyunsaturated fatty acids.

**Table 2:** Subject baseline characteristics ^1^.

	Males (*n* = 21)		Females (*n* = 5)	
Age (year)	57.9	(1.0)	67.4	(2.9)
BMI (kg/m^2^)	29.4	(0.7)	28.4	(0.9)
Waist:hip (cm)	1.0	(0.0)	0.9	(0.0)
Waist:height (cm)	0.6	(0.0)	0.6	(0.0)
SBP (mmHg)	121.2	(2.7)	130.9	(6.5)
DBP (mmHg)	79.6	(1.6)	82.9	(2.1)
Total cholesterol (mg/dL)	192.8	(7.8)	212.4	(6.9)
VLDL (mg/dL)	22.2	(2.8)	20.6	(4.9)
LDL (mg/dL)	120.2	(7.0)	130.6	(7.7)
HDL (mg/dL)	50.2	(2.9)	61.0	(7.5)
TG (mg/dL)	111.9	(14.0)	103.0	(24.5)
Fasting glucose (mg/dL)	98.5	(2.0)	102.6	(3.3)
Fasting insulin (µIU/mL)	13.1	(1.7)	11.9	(2.7)

^1^ Values are means (SEM). Abbreviations: BMI, body mass index; SBP, systolic blood pressure; DBP, diastolic blood pressure; VLDL, very low-density lipoproteins; LDL, low-density lipoproteins; HDL, high-density lipoproteins; TG, triglycerides.

**Table 3:** Mean changes in cardiometabolic risk factors following 4 week intervention with control diet vs. pecan-rich diet (*n* = 26).

	Within Group Differences		Between Group Differences	
	Baseline Mean (SE)	Change with Pecan Diet (SE)	Change with Control Diet (SE)	Difference in Mean Change (SE)
Insulin (µIU/mL)	12.87 (1.43)	−1.31 (0.80)	0.69 (0.80)	−2.00 (0.83) ^1^
Glucose (mg/dL)	99.31 (1.73)	−0.97 (1.74)	0.53 (1.74)	−1.50 (2.33)
HOMA-IR	3.20 (0.37)	−0.31 (0.23)	0.20 (0.23)	−0.51 (0.23) ^1^
HOMA-β	129.32 (15.12)	−15.77 (6.90) ^1^	5.57 (6.89)	−21.34 (8.62) ^1^
Total cholesterol (mg/dL)	196.58 (6.6)	−0.98 (4.13)	7.91 (4.12)	−8.89 (4.41) ^2^
VLDL (mg/dL)	21.88 (2.44)	0.12 (1.38)	0.69 (1.38)	−0.57 (1.90)
LDL (mg/dL)	122.19 (5.87)	−1.22 (3.56)	6.19 (3.55)	−7.41 (3.85) ^2^
HDL (mg/dL)	52.31 (2.79)	0.56 (1.18)	1.46 (1.18)	−0.90 (1.49)
TG (mg/dL)	110.15 (12.08)	0.26 (6.88)	3.17 (6.89)	−2.91 (9.37)
SBP (mmHg)	123.08 (2.55)	−0.84 (1.94)	−0.35 (1.94)	−0.49 (2.43)
DBP (mmHg)	80.23 (1.39)	−1.27 (1.22)	−0.79 (1.22)	−0.48 (1.47)

^1^ *p* < 0.05; ^2^ *p* < 0.07. Abbreviations: SE, standard error; HOMA-IR, Homeostasis Model of Assessment for insulin resistance; HOMA-β, Homeostasis Model of Assessment for beta-cell function; VLDL, very low-density lipoproteins; LDL, low-density lipoproteins; HDL, high-density lipoproteins; TG, triglycerides; SBP, systolic blood pressure; DBP, diastolic blood pressure.

**Table 4:** Mean changes in cardiometabolic risk factor *z*-scores following 4 week intervention with control diet vs. pecan-rich diet (*n* = 26).

	Pecan Diet	Control Diet	Difference in Mean Change	Between Group *p*-Value
*z*_Insulin	−0.41	0.21	−0.62	0.024
*z*_Glucose	−0.11	0.06	−0.17	0.52
*z*_HOMA-IR	−0.36	0.23	−0.59	0.037
*z*_HOMA-β	−1.29	0.45	−1.74	0.021
*z*_Total cholesterol	−0.04	0.31	−0.35	0.056
*z*_LDL	−0.12	0.61	−0.73	0.067
*z*_HDL	0.05	0.14	−0.09	0.55
*z*_Triglycerides	0.01	0.08	−0.07	0.76
Aggregate *z*-score *	−0.14	0.25	−0.39	0.009

* Includes *z*_Insulin, *z*_Glucose, *z*_Total cholesterol, *z*_LDL, and *z*_Triglycerides. Abbreviations: HOMA-IR, Homeostasis Model of Assessment for insulin resistance; HOMA-β, Homeostasis Model of Assessment for beta-cell function; LDL, low-density lipoproteins.

**Table 5:** Mean changes in levels of antioxidant compounds, markers of antioxidant activity, oxidative stress, inflammation and endothelial function following 4 week intervention with control diet vs. pecan-rich diet (*n* = 26).

	Within Group Differences		Between Group Differences	
	Baseline Mean (SE)	Change with Pecan Diet (SE)	Change with Control Diet (SE)	Difference in Mean Change (SE)
α-Tocopherol (µmol/L)	50.26 (3.62)	−1.14 (1.15)	0.53 (1.15)	−1.67 (1.12)
γ-Tocopherol (µmol/L)	3.48 (0.37)	0.13 (0.27)	0.05 (0.27)	0.08 (0.33)
Total phenolics (µg/mL)	988.22 (18.65)	6.47 (16.49)	8.31 (16.01)	−1.84 (23.06)
Total thiols (mM)	0.29 (0.02)	0.00 (0.01)	0.00 (0.01)	−0.004 (0.008)
LDLox (lag time min)	102.19 (4.74)	0.96 (3.39)	0.14 (3.34)	0.82 (3.88)
hsCRP (mg/L)	3.95 (0.97)	0.44 (0.90)	0.13 (0.90)	0.31 (1.27)
E-selectin (ng/mL)	29.92 (2.09)	1.97 (0.80) ^1^	1.54 (0.79) ^2^	0.43 (1.12)
Endothelin-1 (pg/mL)	1.86 (0.17)	0.06 (0.12)	−0.05 (0.12)	0.11 (0.14)

^1^ *p* < 0.05; ^2^ *p* = 0.06. Abbreviations: SE, standard error; LDLox, susceptibility of LDL to Cu+2-induced oxidation; hsCRP, high-sensitivity C-reactive protein.

**Table 6:** Mean changes in levels of predominant red blood cell fatty acids following 4 week intervention with control diet vs. pecan-rich diet (*n* = 26) ^1^.

	Within Group Differences		Between Group Differences	
	Baseline Mean (SE)	Change with Pecan Diet (SE)	Change with Control Diet (SE)	Difference in Mean Change (SE)
SFA	42.90 (0.31)	−0.56 (0.33)	−0.07 (0.33)	−0.49 (0.47)
MUFA	16.41 (0.31)	0.21 (0.12)	−0.25 (0.12)	0.46 (0.16) ^2^
PUFA *n*-6	32.72 (0.45)	0.85 (0.32) ^3^	0.17 (0.32)	0.67 (0.46)
PUFA *n*-3	7.6 (0.32)	−0.29 (0.11) ^3^	−0.26 (0.11) ^3^	−0.03 (0.16)
Palmitic	23.52 (0.18)	−0.32 (0.16)	0.08 (0.16)	−0.40 (0.19) ^3^
Stearic	17.51 (0.26)	−0.30 (0.25)	−0.28 (0.26)	−0.03 (0.37)
Oleic	13.72 (0.29)	0.24 (0.10)	−0.34 (0.09)	0.57 (0.13) ^2^
Linoleic	11.36 (0.27)	0.30 (0.18)	0.04 (0.18)	0.26 (0.25)
Dihomo-Gamma-Linolenic	15.43 (0.39)	0.40 (0.21)	−0.01 (0.21)	0.41 (0.30)
DHA	4.62 (0.26)	−0.17 (0.08)	−0.27 (0.08)	0.10 (0.11)

^1^ Data expressed as molar percentage of fatty acids or mol%; ^2^ *p* < 0.01, ^3^ *p* < 0.05. Abbreviations: SE, standard error; SFA, saturated fatty acids, MUFA, monounsaturated fatty acids; PUFA, polyunsaturated fatty acids; DHA, docosahexaenoic acid.

**Table 7:** Subgroup analyses: Mean changes in cardiometabolic risk factors following 4 week intervention with control diet vs. pecan-rich diet (*n* = 26).

	Change with Pecan Diet	Change with Control Diet	Difference in Mean Change	*p*-Value
Insulin (µIU/mL)				0.09 ^1^
Males (*n* = 21)	−1.50	1.13	−2.63	0.006
Females (*n* = 5)	−0.44	−1.34	0.90	0.63
HOMA-IR				0.09 ^1^
Males (*n* = 21)	−0.36	0.33	−0.69	0.009
Females (*n* = 5)	−0.06	−0.38	0.32	0.54
LDL cholesterol (mg/dL)				0.035 ^1^
Glucose, ≤100 mg/dL (*n* = 13)	5.01	3.98	1.03	0.84
Glucose, >100 mg/dL (*n* = 13)	−7.19	8.39	−15.58	0.006
Triglycerides (mg/dL)				0.041 ^1^
Glucose, ≤100 mg/dL (*n* = 13)	−14.32	8.31	−22.63	0.09
Glucose, >100 mg/dL (*n* = 13)	14.44	−1.76	16.20	0.21
α-Tocopherol:Total cholesterol				0.08 ^1^
Glucose, ≤100 mg/dL (*n* = 13)	−0.52	−0.23	−0.29	0.41
Glucose, >100 mg/dL (*n* = 13)	0.07	−0.56	0.63	0.08
Total cholesterol				0.06 ^1^
LDL, <130 mg/dL (*n* = 16)	4.15	17.20	−13.05	0.035
LDL, ≥130 mg/dL (*n* = 10)	−7.40	−14.20	6.80	0.38
HOMA-β				0.037 ^1^
SBP, <120 mmHg (*n* = 11)	−15.47	−16.55	1.08	0.92
SBP, ≥120 mmHg (*n* = 15)	−15.66	19.91	−35.57	0.003

^1^ *p*-value assessing treatment effect modification. Abbreviations: Abbreviations: HOMA-IR, Homeostasis Model of Assessment for insulin resistance; LDL, low-density lipoproteins; HOMA-β, Homeostasis Model of Assessment for beta-cell function; SBP, systolic blood pressure.
